# Cathodic Blister Evolution in Multilayer Coatings: A Critical Review of Diffusion, Fracture Coupling and Stability Criteria

**DOI:** 10.3390/ma19143084

**Published:** 2026-07-17

**Authors:** Muhammad Qasim Shah, Zulfiqar Ahmad Khan, Adil Saeed, Yonggang Meng

**Affiliations:** 1 NanoCorr, Energy and Modelling (NCEM) Research Group, School of Computing and Engineering, Bournemouth University, Poole BH12 5BB, UKasaeed4@bournemouth.ac.uk (A.S.); 2 State Key Laboratory of Tribology in Advanced Equipment, Tsinghua University, Beijing 100084, China; mengyg@tsinghua.edu.cn; 3 Department of Mechanical Engineering, New Uzbekistan University, Tashkent 100007, Uzbekistan

**Keywords:** tribology, friction, wear, residual stress, diffusion-induced stress, coating delamination, meso-mechanics, Khan–Nazir models, blistering

## Abstract

Tribological systems involving rolling and sliding contacts generate coupled mechanical interactions that govern friction, wear, and surface degradation. These interactions produce multiaxial residual stresses that influence crack initiation, accelerate wear, and promote environmentally assisted damage. In corrosive environments, tribo-corrosion further intensifies material degradation through the combined action of mechanical wear and electrochemical reactions. Protective organic and metallic coatings are widely used to mitigate these effects; however, their performance depends on adhesion, stress evolution, and resistance to coupled mechanical and chemical degradation. Among the principal failure mechanisms, cathodic blistering is strongly influenced by diffusion, interfacial stresses, and tribological loading. This review therefore links cathodic blister evolution with coating degradation under combined tribological and corrosive conditions. The review critically examines the Khan–Nazir meso-mechanics Models I, II, and III, which integrate stress-assisted diffusion, residual stress development, mixed-mode fracture, and coating–substrate delamination. Recent developments have extended these models through substrate deformation, multilayer coating architectures, and electro-chemo-mechanical phase-field simulations. The models demonstrate how diffusion-induced and residual stresses interact with tribological loading to initiate and propagate interfacial defects. The analysis shows that blister evolution is primarily governed by elastic modulus mismatch and friction-induced stress fields, while stability criteria predict non-axisymmetric blister morphologies associated with buckling and delamination. Overall, this review highlights the significance of the Khan–Nazir models for understanding wear, friction, and coating durability in engineering systems. The unified framework provides valuable guidance for the design and optimisation of advanced multilayer protective coatings for marine, automotive, energy, and manufacturing applications operating under rolling/sliding contact and tribo-corrosion environments.

## 1. Introduction

The systems involved in tribology that include rolling and sliding contacts experience frictional heating, surface damage, and gradual wear. The application of mechanical force repeatedly results in rolling contact fatigue (RCF), micro-cracking, and wear-corrosion, which together will reduce the quality of metals in marine, humid and chemically active places [1,2,3,4]. The interaction of these mechanisms results in the formation of substantial residual stress fields that will change with the cyclic loading and lead to the initiation of cracks and the degradation of the surface over a long period [5,6]. Previous research in tribology on ceramic rolling elements has shown that the formation of ring cracks, tensile residual stresses, and RCF-induced stress variations control surface failure during sliding and rolling [7,8]. The findings of such studies also pointed out that the integrity of the surface under tribological load is highly influenced by the stability of residual stress and its interaction with wear and frictional forces [9,10,11].

To counteract these mechanisms of degradation caused by tribology, various coatings such as organic, hybrid, and nanocomposite have been employed as protective layers. The performance of these coatings is governed by factors like adhesion strength, stress accommodation, and resistance to the combined effect of mechanical and chemical attack. The most recent studies have pointed out that there is a growing need for nanocomposite coatings that possess not only a high resistance to wear but also an excellent coating–substrate adhesion that can prevent ultimate blistering [1,5]. Recent advances have further demonstrated that zinc–polymer composite coatings enhanced with nanoparticles, conducting polymers, metal–organic frameworks (MOFs), and two-dimensional materials can significantly improve corrosion resistance, self-healing capability, and long-term durability in aggressive marine environments [11]. The failure of coatings due to tribological loading results in the development of residual stresses at both the surface and subsurface. Earlier research pointed out that rolling and sliding give rise to the generation of tensile and compressive residual stress fields, which in turn promote crack propagation and material fatigue [7,8]. The approach of applying real-time 3D finite element simulations has led to the revealing of how residual cyclic friction (RCF) stress distributions change in time depending on wear, lubrication conditions, and material response [9]. On the other hand, studies on joint friction and dynamic tribological systems have pointed out the role of friction-induced stress variations in maintaining the integrity of the surface [10]. Recent reviews have also highlighted that the tribological performance of protective coatings is strongly governed by coating adhesion, stress accommodation, hardness, stiffness, and resistance to the combined effects of mechanical wear and chemical attack. Moreover, rolling and sliding contacts generate complex residual stress fields that promote crack propagation and material fatigue, making accurate predictions of coating degradation under service conditions particularly challenging [10,11].

Previous tribology experiments have established that the contact points under rolling or sliding produce specific residual stress fields that dictate the start of micro-cracking and the wear of the material through RCF [7,8]. Research has shown that before any sign of wear appears, defects such as ring cracks, regions of tensile stress concentration and cyclic stress redistribution are seen, and these defects are indications of the typical residual-stress behaviour of the sliding surfaces. The previous studies indicate that these mechanical stresses in the subsurface layers under rolling contact with heavy loads can cause nano-crystallisation and grain refinement, thus affecting the properties of the material, such as hardness, ductility, and fatigue resistance. Also, operating at high temperatures can induce thermal softening or expansion, which would in turn affect the stress distribution and accelerate microstructural changes. Together, these factors influence subsurface fatigue, crack initiation and the long-term degradation of mating components [9,10,11]. The pattern of residual stress forms the mechanical basis for the later meso-mechanics formulations, as the stress components that prevail in RCF also determine the diffusion paths, the interfacial energy release and the damage build-up in the coating–substrate systems. Therefore, the subsequent Khan–Nazir Models I–III grasp the earlier residual-stress mechanisms and combine them in a fully coupled diffusion–stress–fracture framework to describe blister evolution [2].

The studies have already established a foundation, namely the residual stress baseline, which is a prerequisite for any discussion around cathodic blistering to be conducted. The formation of blisters is linked with mechanical stresses, the transport of environmental species, and the occurrence of interfacial reactions. Water, ions, and oxygen are the species that the coatings allow passage to and during this process, hydroxyl ions and corrosion products are produced. The chemical expansion caused by water and ions creates both diffusion-induced and residual stresses, stresses that lead to blistering eventually [12,13,14,15,16,17,18]. The combination of diffusion, stress build-up, and mixed-mode fracture is the one that decides the blister’s initiation and growth; the mechanism has been under study for over thirty years now, but it is gaining deeper relevance when looked at through the lens of tribology-driven stress evolution.

Earlier blister studies relying on empirical relationships, such as blister radius vs. adhesion strength, provided qualitative insight [12,13,14] but lacked integration with the mechanical foundations established by tribology, RCF, and residual stress research. This review bridges that gap by linking blistering to the broader tribological stress landscape. These techniques granted qualitative insights, which were of great value in comprehending the impact of temperature, pH, and coating permeability, yet they did not possess the foundation of mechanics. Later fracture-based approaches, influenced by classical interface fracture mechanics frameworks established in earlier thin-film delamination studies [15,17], characterised the cracking process with higher precision, allowing toughness through the interface, energy release rates, and mixed-mode loading during blister propagation to be evaluated. Mechanics-based models derived from classical plate-bending theory [15,16,17,18,19] have been among the most influential developments, providing predictive relations for blister pressure, curvature, and energy release rates. However, these methods mostly viewed blister pressure as a given input instead of a variable produced by the linked diffusion and chemical thermodynamic processes, which restricted their ability to predict.

A major move towards the completely coupled diffusion–mechanics model has been proposed. Their meso-mechanics-based formulation helped synthesise:

A bilayer residual stress model.

Stresses resulting from diffusion that have been chemically induced.

The adaptation of diffusion-induced stresses as an effective blister pressure.

The use of mixed-mode fracture mechanics to predict the behaviour of blisters.

The application of this framework revealed the phenomenon of blistering expanding to be primarily dependent on the modulus ratio, whereas the thickness ratio has a surprisingly tiny influence; this matches the experimental results [2,14,20,21].

Notwithstanding all this, there are still some significant limitations that persist. The stress-coupled chemical potential used in earlier formulations was based on classical expressions for diffusion in stressed solids, which did not include a complete thermodynamic expansion incorporating hydrostatic stress coupling, mechanical work of insertion and configurational entropy [20,21].

The residual-stress analysis ignored higher-order curvature effects and shifts in the neutral axis, whereas the chemical-eigenstrain method for diffusion-induced stress has not been fully derived from the conditions of mechanical equilibrium and compatibility across the coating thickness [22,23,24,25,26]. Furthermore, the model considered only axisymmetric blister growth and thus neglected non-axisymmetric perturbations and telephone-cord instabilities that have been observed in thin-film buckling [27,28,29,30,31,32]. Moreover, the literature is devoid of thorough disclosure of the modelling assumptions, boundary conditions, and computational strategies, which constrain both the reproducibility of the model and its potential for further development.

Researchers have made important progress in coating degradation studies since 2016 by using new electrochemical and mechanical methods, which go beyond existing diffusion-based research methods. The meso-mechanics-based cathodic blister models, which include diffusion-based eigenstrain effects and interfacial fracture properties, explain that blister formation and development depend on the combined effect of coating elastic properties, coating thickness, and residual stresses, together with the strength of coating–substrate interface bonds [2,4]. The predictive capabilities for current coating systems have been improved through model extensions, which emerged after 2016 and included three new elements: substrate plastic deformation, multilayer architectures and electro-chemo-mechanical phase-field simulations [3,5]. Recent research studies demonstrate that coating defects and substrate plastic deformation, together with multilayer architectural elements, determine how stress redistributes throughout coated systems and their risk of developing blisters [3,5]. The 2016 meso-mechanics framework still stands as the main analytical blister model because no new fundamental models have emerged since its creation. The post-2016 advancements to the system extended its ability to forecast outcomes with contemporary coating materials. More recently, electro-chemo-mechanical phase-field formulations have integrated electrolyte transport, interfacial electrochemical behaviour, and mechanically assisted corrosion into a unified computational framework, enabling more realistic simulation of film rupture, dissolution, repassivation, and localised corrosion processes [32]. The electro-chemo-mechanical phase-field modelling development has produced new computational methods which enable researchers to model corrosion damage and interfacial degradation in coating–substrate systems which experience both chemical and mechanical forces [4].

Research into polymer mechanics and polymer microstructure has discovered that multiple stress redistribution mechanisms, which operate at different length scales, will increase resistance against mechanical damage in materials. Xu et al. showed that hydrogen-bonding networks in polyurea systems create hierarchical structures, which enable materials to achieve higher strength and toughness through controlled stress dissipation [1]. The stabilisation of lamellar polymer crystal nuclei establishes control over microstructural development and stress distribution, while it serves as a molecular mechanism that determines how polymer coatings will develop blistering and cracking resistance [6]. The research team demonstrated through their study that lamellar polymer crystal nucleus stabilisation serves as a central mechanism which determines how polymers will organise their structure and display mechanical properties, while demonstrating that microstructural changes impact how stress will be absorbed and materials will resist damage in polymer-based materials [6]. These insights are directly relevant to modern blister- and crack-based coating failure models, which increasingly require accurate representation of stress redistribution and damage tolerance within polymer coating layers. Although this review primarily focuses on cathodic blister evolution in multilayer coatings, it also considers tribological loading, wear, and corrosion because these mechanisms interact under practical service conditions to influence blister initiation, propagation, and coating failure. This integrated perspective provides a more comprehensive understanding of coating degradation and durability.

### 1.1. Scope and Contributions of the Present Review

The updated research provides a modern, fully derived meso-mechanics model addressing these limitations. Key achievements include:

- The comprehensive derivation of the stress-coupled chemical potential beginning from configurational thermodynamics [17,18,22];
- Developing a fully derived stress-assisted diffusion equation that ties together hydrostatic stress, partial molar volume, and concentration gradients [23,24,25];
- The formulation of a modern bilayer residual-stress model that considers curvature, elastic mismatch, and neutral-axis shifts [14,21,23];
- A detailed derivation of diffusion-induced stresses through the application of chemical eigenstrain and mechanical equilibrium [24,25,26];
- A universal pressure evolution model that translates diffusion-induced stresses into an effective blister pressure through a consistent mechanical–chemical feedback mechanism [2,20,22];
- An advanced fracture-mechanics formulation that combines the von Kármán plate theory with the mixed-mode dependent energy release rates and the interface toughness [15,16,17,22,23];
- A new stability framework that predicts both circular and non-axisymmetric blister transitions [27,28,29,30,31,32].

The methodology incorporates the governing equations, boundary conditions, numerical schemes, and validation protocols. The resulting framework provides a physically consistent, predictive description of cathodic blister initiation, growth, and stability. It enhances the mechanistic understanding of coating degradation and provides a quantitative outlook on the impacts of modulus ratio, thickness ratio, partial molar volume, and environmental conditions. Furthermore, it creates a robust base for future three-dimensional, time-dependent, and environment-adaptive coating degradation models.

### 1.2. Post-2016 Extensions of the Meso-Mechanics-Based Blister Model

Table 1 summarises key post-2016 developments in meso-mechanics-based blister modelling and related polymer coating studies, highlighting advances in substrate deformation, multilayer architecture, phase-field simulations, and microstructural polymer mechanisms. The additional studies demonstrate how environmental conditions, mechanical deformation and prognostic modelling approaches affect the process of blister initiation and growth.

The table presents essential advancements that allow researchers to expand the 2016 meso-mechanics model through the implementation of substrate deformation and multilayer architecture, phase-field simulations and polymer microstructural mechanisms. The additional studies demonstrate how mechanical deformation, environmental conditions and predictive or prognostic modelling approaches affect the processes of blister nucleation and growth and coating failure.

## 2. Scientific Background and Review of Coupled Degradation Mechanisms

Blistering is unquestionably one of the major defects that lead to the failure of organic coatings on metals. This phenomenon is manifested as small, localised dome-shaped areas where the coating film is detached. The detachment is produced by a couple of combinations of chemical, mechanical, and interfacial processes. Therefore, a clear understanding of the historical development of models for these individual mechanisms for the development of a physically consistent meso-mechanics-based blister-growth model is essential.

**Figure 1:**
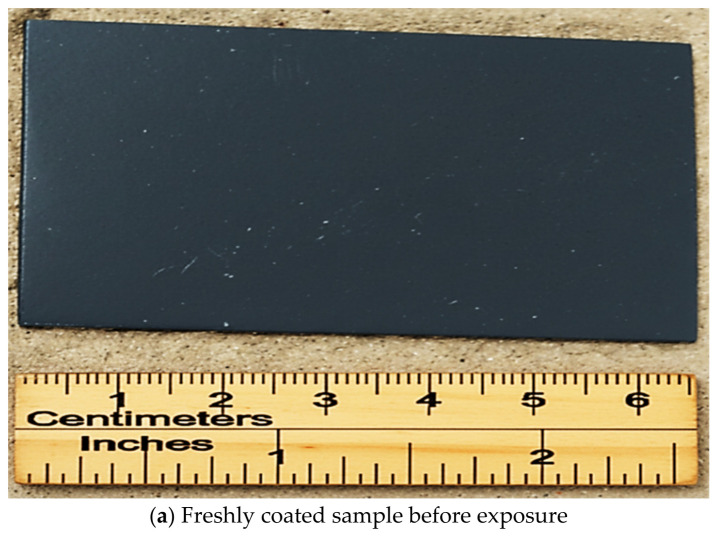

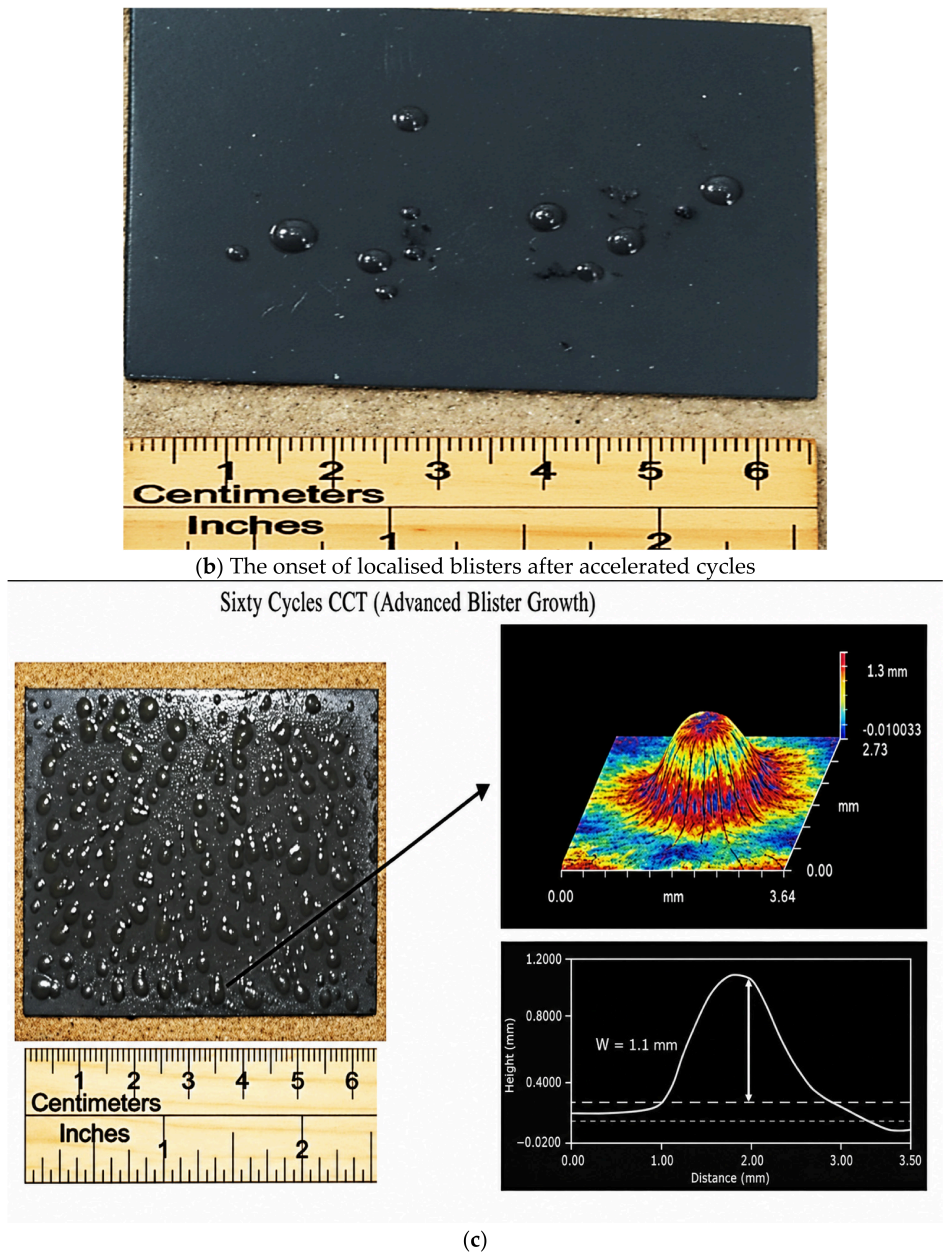
Sequential blistering of a coated metallic substrate in cyclic corrosive exposure: (**a**–**c**) [1]. (**c**) Advanced blister growth and increased density after 60 cycles, with an inset showing a 3D surface profile and cross-section of a typical blister.

The evolution of blistering on a coated metallic substrate in corrosive environments is shown in Figure 1 [1], which traces the sequence of events from an initial intact state to extensive localised degradation.

### 2.1. Initiation Mechanisms and Driving Forces (Diffusion and Osmosis)

The section concentrates on the start-up processes of coating blistering from the viewpoint of tribology, thus paving the way for gear tribology studies. In gears, rolling and sliding contacts produce residual stress that promotes cracks and surface deterioration due to fatigue. A mechanistic understanding of the diffusion and osmosis-driven processes in coatings before one can predict the material response to cyclic loading. Even though this review covers the basic mechanisms, computational simulations, and advanced modelling techniques for gear materials will be the subject of later papers.

The first step in blister formation normally takes place when the coating material is invaded by the aggressive species, which are, at most, water, dissolved oxygen, chloride ions, or products from the cathodic reaction. These species, after penetrating the coating material, accumulate at the interface between the coating and the substrate. The penetration of aggressive species can be through various channels such as micropores, defects, and bulk diffusion/capillary pathways [1,2,3,4]. When soluble corrosion products and electrochemically generated species (for example, OH^−^) get trapped under the coating, they create osmotic and chemical pressures; the resulting osmotic and chemical pressure gradients lift the coating and facilitate the interfacial concurrent processes of coating detachment and degradation [5,6,7,8,9,10,11].

In the beginning, diffusion models relied on Fickian mathematical formulations to portray the evolution of concentration in coatings. Subsequently, thermodynamic analyses demonstrated that chemical potential and therefore mass transport are modified by elastic stresses, requiring stress-coupled diffusion equations for accurate prediction of species transport in constrained films [24,25,26]. The modern formulations involve hydrostatic stress, partial molar volume (or insertion volume), and concentration-dependent diffusivity to obtain the entire picture of the mechanical feedback on transport.

### 2.2. Evolution of Stress and Bilayer Mechanics

In gear systems and other tribological contacts, the growth of blisters is strongly influenced by residual stresses that develop under repeated rolling and sliding loads. These stresses interact with fatigue mechanisms, leading to crack initiation and surface degradation in coated materials. Understanding bilayer mechanics and stress evolution provides essential insight for predicting material performance under operational tribological conditions, setting the stage for simulations and numerical modelling in gear tribology studies.

After the first stage of blistering, the growth of the blister is determined by the mechanical state of the coating, which is controlled by residual stresses and the volumetric changes brought about by the ingress of species. The stresses that remain after the curing process cause the coating to shrink; they depend on the residual stresses that thermal expansion mismatch and deposition processes create and can counteract or accelerate interfacial failure depending on their direction and magnitude. Diffusion-induced stresses give rise to local volumetric expansions (chemical eigenstrain) and can dominate the stress state in thin, flexible coatings that are constrained by much stiffer substrates [6,7,8,9,10,11,24,25,26].

Theoretical approaches based on beam and plate models for classical bilayer systems, dealing with the thermal mismatch, give the first and primary expressions for the distribution of stress through the thickness and the bending of coated materials [15,16,17,18,19]. Subsequent studies show that these concepts were transferred to various kinds of terrains, namely, the polymeric and hybrid coatings; recent studies highlighted the importance of higher-order curvature and neutral axis shift effects, which in turn resulted in significantly altered stress gradients and bending moments in the case of real coated systems [39,40,41,42,43].

### 2.3. Interfacial Delamination and Fracture Processes

In gears and other parts subject to friction, often due to rolling and sliding contact, fracture is the ultimate result of fatigue. Material imperfections combined with the residual stresses from the repeated loading create conditions that lead to the delamination of the interface and the propagation of the crack. It is crucial to first discuss the blister mechanics before delving into the background of rolling contact fatigue and its role in driving fracture in coated tribological systems [2,3,4]. With the growth of blisters, there is a gradual rupture of the bond between the coating and the substrate. The delamination process is furthered when the energy rate of blister growth surpasses the interfacial toughness. Hutchinson and Suo, along with others, have brought up thin-film interface fracture mechanics that give mixed-mode (Modes I/II) criteria for determining the energy release rates as well as the mixed-mode for the interfacial cracks. Thus, it opens the avenue for judging interfacial toughness and the direction of crack propagation under the application of combined opening and shear load components [13,14,15].

The work concerning the circular blister shape (plate bending due to internal pressure) gave a detailed account of the relations existing amongst the internal pressure, the curvature of the film, and the rates of energy release; the latter being an area of study that forms the backdrop for many mechanics-based blister models [16,17,18,19]. When blisters are large and compressive residual stress is higher, the instability in the geometry might turn into telephone-cord buckling or spiral morphologies, all that is determined by the interplay of the internal pressure, film bending rigidity, and interfacial resistance; the experimental and numerical studies of telephone-cord buckling have revealed the role of various factors like imperfections, wavelength selection, and propagation criteria in these morphologies [27,28,29,30,31,32].

### 2.4. Limitations of Current Meso-Mechanics Models

Models of blistering based on mechanics that were made before Khan–Nazir’s models [20,40,41,42] succeeded in illustrating the most important geometric and energetic trends, but not always with complete thermodynamic rigour and a full account of the diffusion/stress interaction. The Khan–Nazir Models [20,40,41,42] merged diffusion-induced stress, bilayer mechanics, and mixed-mode fracture and have taken a considerable step toward the predictive interlinking of transport and mechanics in blister growth; however, significant simplifications still exist. The simplifications included a partially asserted (rather than fully derived) stress-coupled chemical potential, simplified chemical eigenstrain assumptions, and restriction to axisymmetric blister morphologies [20,40,41,42].

The classical cathodic blister models treat the coating layer as an elastic or viscoelastic material which behaves uniformly throughout its entire volume. The meso-mechanics-based diffusion–fracture formulations, which combine ionic transport with interfacial crack propagation mechanisms [2], offer better prediction capacity. Recent studies have also emphasised that coating defects, interfacial adhesion, electrolyte transport, and localised electrochemical reactions play a critical role in the initiation and propagation of cathodic delamination under service conditions [44,45]. Current electro-chemo-mechanical phase-field models have improved predictive accuracy through their ability to simulate damage initiation and progression during simultaneous chemical and mechanical testing, yet they cannot model molecular- and microstructural-scale polymer network toughening mechanisms [4].

However, recent polymer mechanics studies, such as those by Xu et al., indicate that hierarchical hydrogen-bonding architectures in polyurea systems enable controlled activation of sacrificial bonds, leading to enhanced toughness and delayed crack propagation under mechanical loading [1]. The research conducted by Xu et al. about the stabilisation of lamellar polymer crystal nuclei demonstrates that polymer microstructural evolution determines how stress distributes and how mechanical properties respond to external forces [6]. Moreover, recent studies on coating defects, substrate deformation, and multilayer coating designs demonstrate that tailored architectures and graded material properties can reduce diffusion-induced pressure accumulation and suppress crack initiation, underscoring the need for comprehensive electro-chemo-mechanical models that account for coating architecture, material nonlinearity, and substrate constraint effects [3,5]. Recent advances in nanocomposite organic coatings have further demonstrated that nanomaterials enhance the barrier properties of protective coatings by reducing porosity and increasing diffusion tortuosity, thereby improving corrosion resistance and coating durability. Moreover, smart nanocomposite coatings and multiscale simulation approaches are emerging as promising strategies for evaluating long-term anticorrosion performance [46].

Overall, the advancements found in the literature are quite impressive, yet there is still a gap: a unified, entirely derived framework that precisely incorporates configurational thermodynamics, computes diffusion-induced stresses across bilayer thicknesses, translates stress feedback into an emerging blister pressure, and anticipates both axisymmetric and non-axisymmetric blister stability still requires construction.

## 3. Analytical Framework and Theoretical Model

**Figure 2:**
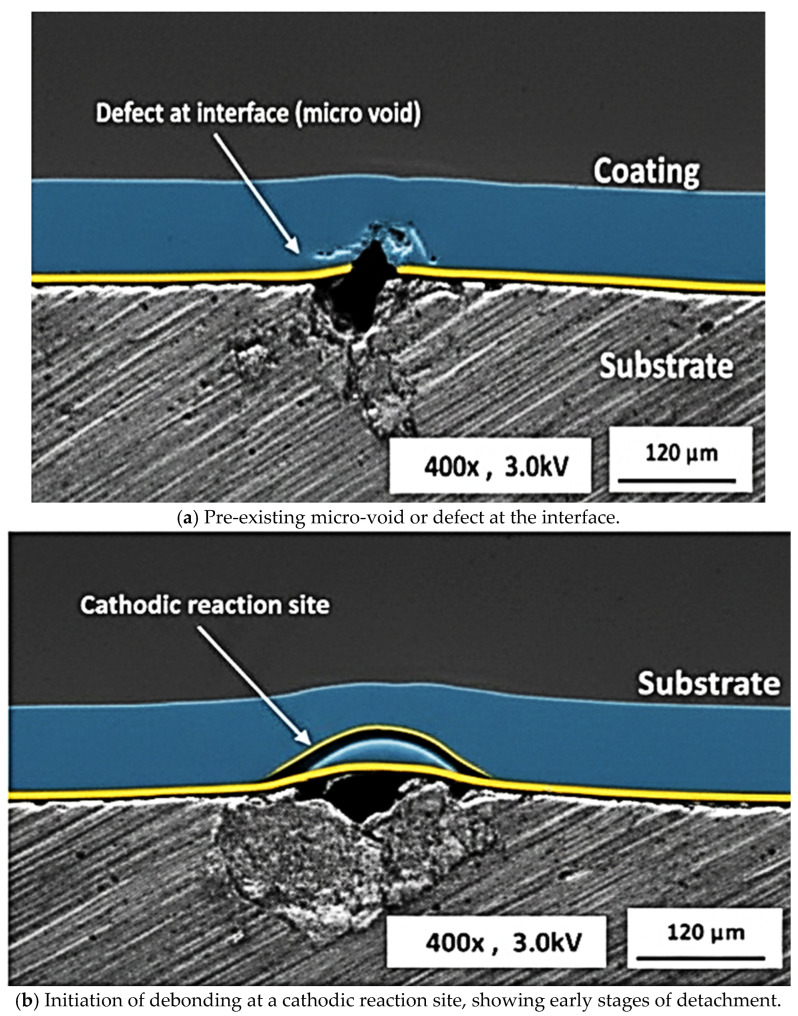

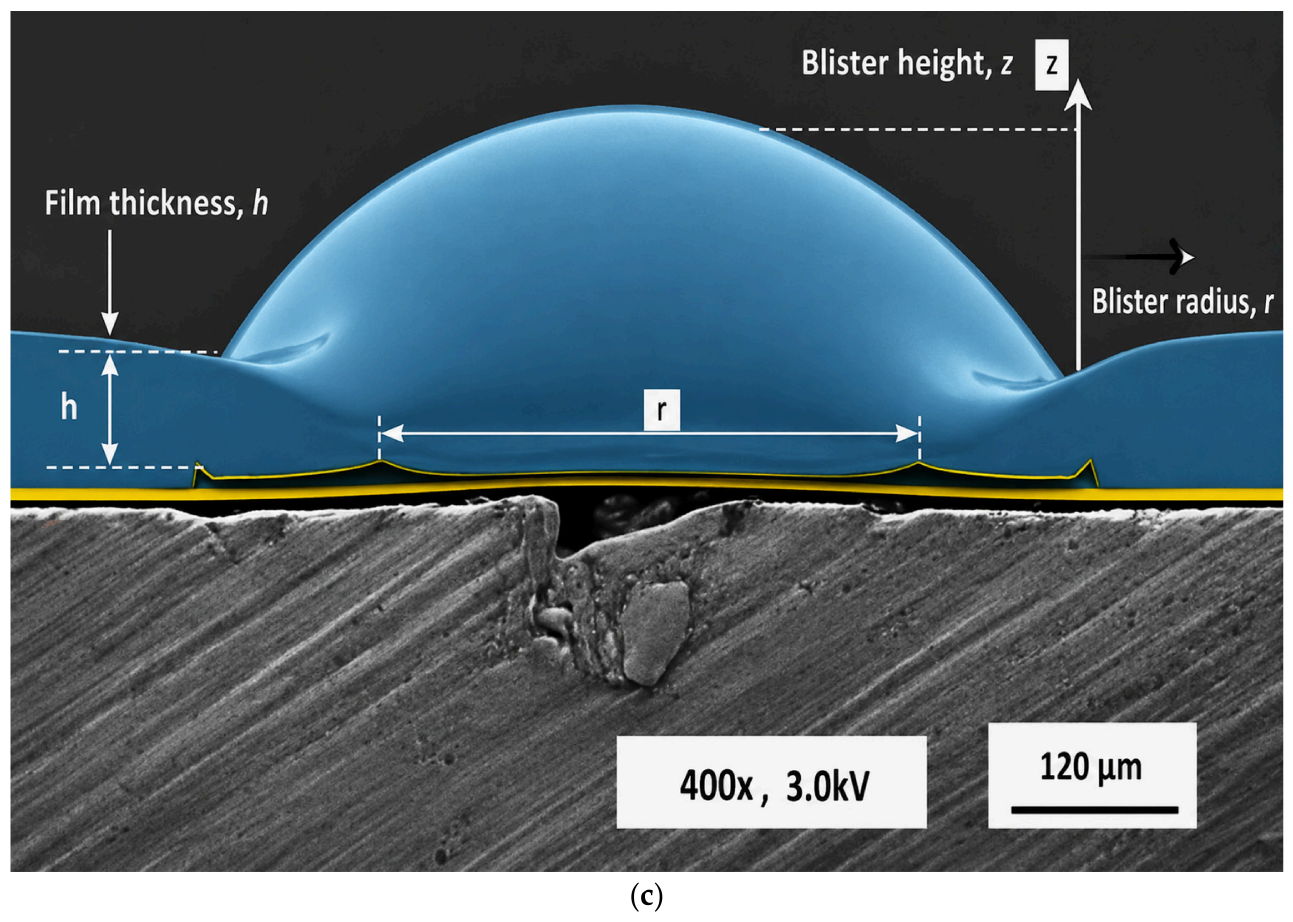
Scanning Electron Microscopy (SEM) cross-sectional analysis illustrating key stages and features of blistering at the coating–substrate interface: (**a**–**c**) [1]. (**c**) Formation of a circular blister, visually defining key geometric parameters such as blister height (z) and radius (r) used in the analytical model.

The new model presented is the result of a series of clearly defined assumptions intended to facilitate analytical treatment while at the same time providing the necessary physical consistency. Microscopic research plays an important role in getting to understand the processes leading to blister formation and the resulting geometry. Representative SEM cross-sections illustrate pre-existing interfacial defects, cathodic reaction spots, which are places where debonding starts, and the typical geometry of existing blisters are the main features; images identify the key geometric parameters used in the analytical model, including the blister height (z), blister radius (r), and film thickness (h), as shown in Figure 2 [1].

### 3.1. Model Assumptions

The coating and the substrate are considered linear elastic, isotropic materials with constant layer properties. The layers are bonded tightly before separating; under the thin-film approximation, the lateral gradients of diffusion are considered negligible; species entry-induced chemical expansion is modelled by a volumetric eigenstrain; thermal mismatch and deposition create residual stresses; diffusion occurs through a stress-assisted Fickian (Onsager-type) mechanism; and the main pathway for transport is through the coating thickness. Blisters are considered the result of interfacial debonding subjected to a combination of residual and diffusion-induced stresses, and fracture is studied according to the mixed-mode dependent fracture mechanics. For the quasi-static analysis, environmental conditions are held constant.

### 3.2. Materials Framework

The bilayer system consists of a coating of thickness ℎ, which is a polymer that is glued to a substrate that is metallic and of thickness s. The coating is softer and more prone to chemical attack, while the substrate limits the mechanical deformation and is modelled as a type that does not allow the passage of substances. The material characteristics that are required are Young’s modulus (Ec, Es), Poisson’s ratio (ν), thermal expansion coefficients (αc, αs), and the partial molar volume of diffusing species (Ωk). The bilayer thickness ratio (h/s) and modulus ratio (Ec/Es) are treated as key variables in parametric studies. Chemical species are diffusive within the coating.

D in the coating is constant for initial studies; further studies change modulus and thickness ratios to find sensitivity [1,2,3,4,5,6,7].

### 3.3. Mathematical Modelling Workflow

The work follows a sequential analytical–numerical coupling approach: (1) thermodynamic stress-coupled chemical potential derivation, which includes hydrostatic stress and configurational terms (Larché–Cahn formalism and modern extensions); (2) the formulation of stress-assisted diffusion via mass conservation and the use of Onsager relations; (3) calculation of residual stresses within the bilayer through linear beam/plate theory (midplane strain, curvature, neutral axis); (4) acquisition of diffusion-induced stresses using chemical eigenstrain as an input to the mechanical equilibrium problem; (5) translation of diffusion-induced stresses into an effective blister pressure by means of a reliable mechanical–chemical feedback law; (6) application of von Kármán plate theory and mixed-mode interfacial fracture mechanics to identify energy release rates and conditions for crack propagation; (7) conduction of linear stability analysis for transitions from axisymmetric to non-axisymmetric bifurcations. This workflow not only follows but also broadens the conceptual path previously utilised in meso-mechanics and fracture-coupled diffusion modelling.

### 3.4. Governing Equations Strategy

The governing equations are: (i) stress-coupled diffusion equation for the transport of species; (ii) mechanical equilibrium for the residual and diffusion-induced stresses (linear elasticity, plate bending); and (iii) fracture mechanics relations for interfacial delamination (mode-adjusted energy release rates). The diffusion equation incorporates hydrostatic stress through partial molar volume terms; residual stress is based on the bilayer bending theory; diffusion-induced stress is derived from eigenstrain + compatibility; and the blister propagation criterion is a normalised, mode-adjusted energy release rate that connects diffusion-induced pressure to interfacial toughness.

### 3.5. Coupling Scheme

The coupling is sequential (staggered): diffusion → mechanical solve → pressure conversion → fracture check → feedback to diffusion. This keeps each subproblem easy to manage and enhances numerical stability while accurately capturing the significant nonlinear interactions between transport, stress, and interfacial fracture. The iteration continues at each time step until convergence is reached.

### 3.6. Validation Approach

Validation consists of comparing experimental observations and previously published numerical results; the comparison is made based on the maximum blister height, critical radius, residual stress distribution, and sensitivity to modulus and thickness ratios. Validation of model assumptions comes from the agreement with benchmark cases; discrepancies guide the identification of such higher-order effects (viscoelasticity, plasticity, environmental ageing) that have been missed.

### 3.7. Numerical Method Outline

The coupled system is solved with an implicit, time-marching finite difference scheme across the entire coating thickness (N nodes). Boundary conditions: specified concentration at the exposed surface, no flux at the coating–substrate interface (impermeable), and mechanical boundary conditions for the bilayer edges. Concentration, diffusion-induced stress, feedback pressure, and fracture evaluation are sequentially iterated per time step until convergence is achieved. The independence of the mesh is verified; the different combinations of modulus ratio, thickness ratio, and partial molar volume are among the parametric studies.

## 4. Analytical Framework

Thermodynamic Basis (Chemical Potential → Flux → Diffusion Equation).

Governing Equation for Stress-Coupled Diffusion.

The initial step in understanding blister formation involves the diffusion of aggressive species (e.g., water, ions) through the coating matrix towards the coating–substrate interface. This process is governed by a generalised diffusion equation that accounts for the influence of mechanical stress on the chemical potential, as first rigorously established in earlier thermodynamic formulations of stress-coupled diffusion [24,25]. The total chemical potential $\mu_{\mathrm{total}}$ of the diffusing species (solute k) represents the thermodynamic driving force for mass transport in a stressed solid. It is expressed as the sum of the standard chemical potential $\left(\mu_{k}\right)$ and the mechanical work potential $\left(\mu_{\mathrm{mech}}\right)$ due to the stress field [2,24,25]:

(1) $$\mu_{\mathrm{total}\;}={\;\mu }_{k}\;+{\;\mu }_{\mathrm{mech}}$$

The mechanical contribution, $\mu_{\mathrm{mech}}$, is the partial molar work required to introduce the species into the stressed solid, often expressed by the integrated form adopted in previous stress–diffusion studies [24,25]:

(2) $$\mu_{\mathrm{mech}}\;=\;-{\int_{0}^{\sigma_{i}}\overset{ˉ}{V}}_{k}d\sigma_{i}$$

where d$\sigma_{i}$ = $\left(\frac{1}{3}\int_{i=1}^{i=3}\sigma_{i}\right)$

The sign convention follows the definition of hydrostatic stress adopted in the Khan–Nazir model. Accordingly, the negative sign in the integral formulation is absorbed into the hydrostatic stress definition, yielding the linearised expression in Equation (3).

$$\mu_{\mathrm{mech}}=-{\int_{0}^{\sigma_{i}}\overset{ˉ}{V}}_{k}d\left(\frac{1}{3}\sum_{i=1}^{i=3}\sigma_{i}\right)$$

${\overset{ˉ}{V}}_{k}$ is the partial molar volume of species k.

$\sigma_{i}(i\;=\;1,2,3$) are the principal stresses.

Assuming small elastic strains and a constant partial molar volume $v_{k}$, the integral simplifies to the linearised working form [2,5]:

(3) $$\mu_{\mathrm{mech}}\;={\;V}_{k}\;\sigma_{h}$$

$\sigma_{h}=\frac{1}{3}(\sigma_{1}+\sigma_{2}+\sigma_{3})$ is the hydrostatic stress (mean stress).

**Figure 3:**
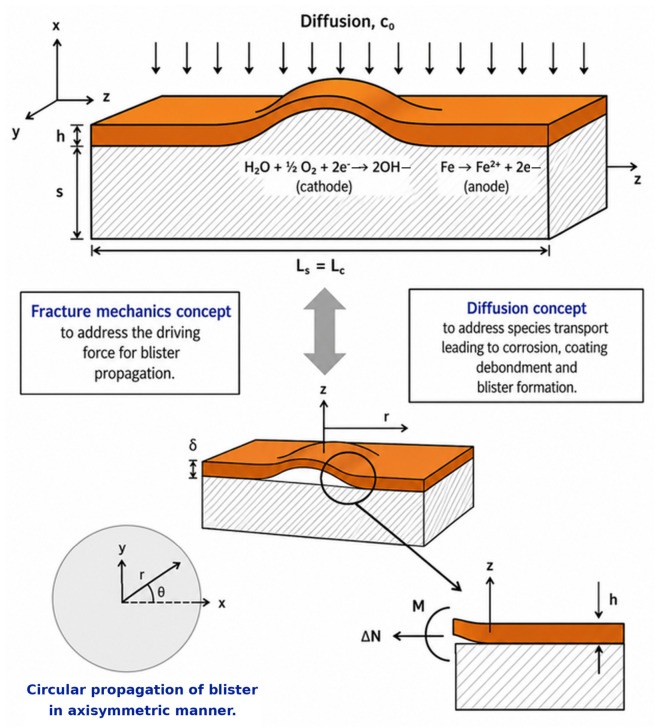
Two approach models of blistering with the diffusion concept (**upper**) coupled with the fracture mechanics concept (**lower**). The double-headed arrow indicates the coupling between species transport and fracture-driven blister propagation [1].

Combining Equation (1) with the ideal dilute solution concentration term, the total potential adopted in the meso-mechanics model by the Khan–Nazir approach, as shown in Figure 3 [1], is:

(4) $$\mu_{\mathrm{total}}\;=\;\mu_{k\;}^{0}+\;\mathrm{RT}\ln C\;+{\;V}_{k}\;\sigma_{h}$$

The term ${V_{k}\sigma }_{h}$ explicitly couples the stress field to diffusion: tensile stress $(\sigma_{h}\;>\;0)$ increases the chemical potential, driving ions toward regions of lower stress (compression). $\sigma_{h}=\frac{1}{3}(\sigma_{1}+\sigma_{2}+\sigma_{3})$ is the hydrostatic stress [5,17].

Flux Law (Linear Onsager Form).

The derivation of the stress-coupled diffusion equation begins with the fundamental Onsager Relation, which states that the flux of a species $(J_{k}$) is proportional to the negative gradient of its total chemical potential $\left(\mu_{\mathrm{total}}\right)$ [2,3,13]

(5) $$J_{k}=-Mc_{k}\nabla \mu_{\mathrm{total}}$$

where M is the mobility, related to the diffusion coefficient $D_{k}$ by the Einstein relation M = $\frac{D_{k}\;}{\mathrm{RT}}$ [13,26,30].

$$J_{k\;}=-\frac{D_{k}\;c_{k}}{\mathrm{RT}}\;\nabla \mu_{\mathrm{total}}\;$$

Substituting the definition of the total potential $\mu_{\mathrm{total}}$ back into the flux expression and then applying the Conservation of Mass principle $(\partial c_{k}/\partial t\;=-\nabla \cdot J_{k})$ yields Equation [25].

The negative sign from the divergence operator in the Conservation of Mass equation $(\partial c_{k}/\partial t\;=-\nabla \cdot J_{k}$) cancels out the negative sign in the generalised flux equation.

Resulting in the final form:

$$\frac{\partial c_{k}}{\partial t}\;=\;\vec{\;\nabla }(\frac{D_{k}\;c_{k}}{\mathrm{RT}}\;\nabla \mu_{\mathrm{total}})$$

(6) $$\frac{\partial c_{k}}{\partial t}=\vec{\nabla }\cdot \left\{\frac{D_{k}c_{k}}{\mathrm{RT}}\left[\mu_{k}-{\int_{0}^{\sigma_{i}}\overset{ˉ}{V}}_{k}d\left(\frac{1}{3}\sum_{i=1}^{i=3}\sigma_{i}\right)\right]\right\}$$

(i = 1,2,3) where $c_{k}$ is the concentration, $D_{k}$ the diffusion coefficient, R the gas constant, T the absolute temperature, and σ the Cauchy stress tensor. This equation quantifies the coupled influence of stress gradients and concentration gradients on diffusion flux within the coating. The second term on the right-hand side represents the stress-assisted diffusion flux that becomes significant in coating–substrate systems under mechanical constraint. Vk is a scalar term excluding the effect of the stress tensor, symbolising the partial molar volume of diffusing species k and obtained using Euler’s first theorem for homogeneous functions.

$$V_{k}={\left(\frac{\partial V_{m_{k}}}{\partial n_{k}}\right)}_{T,\;P,n_{k}}={\left(\frac{V_{m_{k2}}-V_{m_{k1}}}{n_{k2}-n_{k1}}\right)}_{T,\;P,n_{k}\;\neq i}$$

Substituting the definition of the total chemical potential (Equation) and the flux law (Equation) into the Conservation of Mass principle $(\partial c_{k}/\partial t\;=-\nabla \cdot J_{k})$ yields the generalised, three-dimensional stress-coupled diffusion equation [2,30,31].

With respect to the above elastic solid as a double-layer cantilever beam, one coat is subjected to a dispersing species, maintaining a concentration denoted as $c_{0}$. In accordance with the current structural design configuration, the upper stratum of the bilayer cantilever beam acts as a coating.

The diffusion equation for the coating, considering the coupled effects of residual stress $\left(\sigma_{r,c}\right)$ and diffusion-induced stress $\left(\sigma_{d,c}\right)$, is expressed as

$$\begin{array}{cccc} & \frac{\partial c_{k,c}}{\partial t}=D_{k,c}\left\{\nabla^{2}c_{k,c}-\frac{{\overset{ˉ}{V}}_{k,c}}{3RT}\nabla c_{k,c}\cdot \nabla \left[\sum_{i=1}^{i=3}(\sigma_{r_{i}}+\sigma_{d_{i}})\right]-\frac{{\overset{ˉ}{V}}_{k,c}}{3RT}\nabla c_{k,c}\nabla^{2}\left[\sum_{i=1}^{i=3}(\sigma_{r_{i}}+\sigma_{d_{i}})\right]\right\} & & \end{array}$$

Considering the double-layer cantilever beam configuration shown in Figure 3 [1], the upper layer represents the coating (thickness h) subjected to the diffusion of chemical species, whereas the lower layer acts as the substrate. By defining the coating–substrate interface at x=h and the substrate boundary at x=−s, Equation (6) reduces to

(7) $$\frac{\partial c_{k,c}}{\partial t}=D_{k,c}\left\{\nabla^{2}c_{k,c}-\frac{{\overset{ˉ}{V}}_{k,c}}{9RT}\nabla c_{k,c}\cdot \nabla (\sigma_{r,c}+\sigma_{d,c})-\frac{{\overset{ˉ}{V}}_{k,c}}{9RT}c_{k,c}\nabla^{2}(\sigma_{r,c}+\sigma_{d,c})\right\}$$

### 4.1. One-Dimensional Coupled Diffusion in the Coating

For a thin coating of thickness h bonded to a metallic substrate, diffusion is assumed to occur primarily along the coating thickness (x). Neglecting lateral gradients, Equation (7) simplifies to [2,13,30]

(8) $$\frac{\partial c_{k,c}}{\partial t}=D_{k,c}\left[\left(1+\frac{E_{c}V_{k,c}^{2}}{9RT}c_{k,c}\right)\frac{\partial^{2}c_{k,c}}{\partial x^{2}}+\frac{E_{c}V_{k,c}^{2}}{9RT}{\left(\frac{\partial c_{k,c}}{\partial x}\right)}^{2}-\frac{V_{k,c}^{2}}{9RT}\frac{\partial c_{k,c}}{\partial x}\left(\frac{\partial \sigma_{r,c}}{\partial x}+\frac{\partial \sigma_{d,c}}{\partial x}\right)\right]$$

where $\sigma_{r,c}$ denotes the residual stress in the coating, and $\sigma_{d,c}$ represents the diffusion-induced stress. This governing equation describes the evolution of concentration under the combined effects of Fickian diffusion, residual stress, and diffusion-induced stress, thereby capturing the coupled chemo-mechanical behaviour of the coating.

Neglecting lateral gradients, the governing equation simplifies to $\;\begin{array}{cccc} & \frac{\partial c_{c}}{\partial t}=D_{c}\frac{\partial^{2}c_{c}}{\partial x^{2}}-D_{c}\left(\frac{V_{k,c}}{RT}\right)\frac{\partial }{\partial x}\left[c_{c}\left(\frac{\partial \sigma_{r,c}}{\partial x}+\frac{\partial \sigma_{d,c}}{\partial x}\right)\right] & & \end{array}$ where $\sigma_{r,c}\;$ is the residual stress in the coating, and $\sigma_{d,c\;}$ is the diffusion-induced stress in the coating. This shows that the concentration evolution is governed by Fickian diffusion and the gradient of total stress, which provides mechanical feedback to diffusion [1,11,17,18].

### 4.2. Residual and Diffusion-Induced Stresses in a Bilayer Coating–Substrate System

Residual stresses originate from differences in elastic and thermal properties between the coating and substrate during deposition and cooling. For a bilayer cantilever coating thickness h and substrate thickness s), the residual stress is determined by solving the elastic bilayer bending equation [1,17,31,32,47].

The derivation of both residual and diffusion stresses relies on the linear elastic beam theory. The in-plane strain ($\varepsilon_{\mathrm{xx}}$) varies linearly through the thickness [1,31]:

(9) $$\varepsilon_{\mathrm{xx}}\left(x\right)\;=\;\varepsilon_{0}\;+\;\kappa (x-t_{b})$$

where $\varepsilon_{0}$ is the mid-plane strain, κ the curvature, and $t_{b}$ the neutral-axis position. The mechanical stress is defined by the total strain minus the initial strain input ($\varepsilon_{\mathrm{xx}}^{\mathrm{input}}$):

(10) $$\sigma_{\mathrm{xx}}\;=\;E(\varepsilon_{\mathrm{xx}}-\varepsilon_{\mathrm{xx}}^{\mathrm{input}})$$

For a free bilayer with no external load, the stress field must satisfy Force and Moment Equilibrium:

(11) $$\int_{-s}^{h}\sigma_{\mathrm{xx}}(x)\;\mathrm{dx}\;=\;0,\;\int_{-s}^{h}\sigma_{\mathrm{xx}}(x)(x-t_{b})\;\mathrm{dx}\;=\;0$$

Derivation of the Final Expression for $\sigma_{r,c}$

The final residual stress is derived by solving the mechanical framework (Equations (9)–(11)) using the thermal strain (αΔT) as the input $\varepsilon_{\mathrm{xx}}^{\mathrm{input}}$. The final expression is [1,17,31,32]


(12) $$\sigma_{r,c}=E_{c}\left[\left(\frac{E_{s}\alpha_{s}s+E_{c}\alpha_{c}h}{E_{s}s+E_{c}h}\right)\Delta T+\frac{x-t_{b}}{\zeta_{r}}-\alpha_{c}\Delta T\right]$$

Ec, Es = Young’s moduli of coating and substrate,

αc, αs = coefficients of thermal expansion,

ΔT = temperature change

The neutral-axis position ${\;t}_{b}$, which is solved from the equilibrium conditions, is:

(13) $$t_{b}\;=-\frac{E_{s}s^{2}\;+{\;E}_{c}h^{2}}{2(E_{s}s\;+\;E_{c}h)}$$

The stress component relies on the residual curvature $\left(\zeta_{r}\right)$ of the beam, which is derived from the balance of moments and forces in the system.

Residual Curvature $\left(\frac{1}{\zeta_{r}}\right)$

The inverse of the residual curvature $\left(\frac{1}{\zeta_{r}}\right)$ is derived by considering the integrated forces and moments within the two layers:

$$\frac{1}{\zeta_{r}}\;=\;\frac{3\left[E_{s}S^{2}\left(\frac{(E_{s}\alpha_{s}S\;+\;E_{c}\alpha_{c}h)\Delta T}{E_{s}S\;+\;E_{c}h}-\alpha_{s}\Delta T\right)-E_{c}h^{2}\left(\frac{(E_{s}\alpha_{s}S\;+{\;E}_{c}\alpha_{c}h)\Delta T}{E_{s}S\;+{\;E}_{c}h}-\alpha_{c}\Delta T\right)\right]}{E_{s}S^{2}(2s\;+\;3t_{b})\;+\;E_{c}h^{2}(2h-3t_{b})}$$

This expression, or its equivalent simplified form, defines the radius of curvature $\left(\zeta_{r}\right)$ of the bilayer cantilever system is attributed to the residual thermal stress. The position of the bending strain component zero is defined by $t_{b}$. The coupling of the diffusion and mechanical models is achieved by replacing the residual stress $\left(\sigma_{\mathrm{rc}}\right)$ with the sum of residual and diffusion-induced stress $\left(\sigma_{\mathrm{rc}}+\sigma_{\mathrm{dc}}\right)$ in the overall mechanical equilibrium equations:

(14) $$\sigma_{r,c}=-E_{c}\left[\left(\frac{E_{s}\alpha_{s}s+E_{c}\alpha_{c}h}{E_{s}s+E_{c}h}\right)\Delta T+(x-t_{b})\left(\frac{3\left[E_{s}s^{2}\left(\frac{(E_{s}\alpha_{s}s+E_{c}\alpha_{c}h)\Delta T}{E_{s}s+E_{c}h}-\alpha_{s}\Delta T\right)-E_{c}h^{2}\left(\frac{(E_{s}\alpha_{s}s+E_{c}\alpha_{c}h)\Delta T}{E_{s}s+E_{c}h}-\alpha_{c}\Delta T\right)\right]}{E_{s}s^{2}(2s+3t_{b})+E_{c}h^{2}(2h-3t_{b})}\right)-\alpha_{c}\Delta T\right]$$

This expression defines the through-thickness variation in residual stress $\sigma_{r_{c}}\left(x\right)$, which can switch sign from tensile to compressive depending on material and geometric parameters.

### 4.3. Stress-Coupled Diffusion Equation

The final form of the stress-coupled diffusion equation (Equation (8)) used in this analysis incorporates the specific geometric and residual strain $\left(\varepsilon \right)\;$ boundary conditions of the bilayer system [1,41,48], extending Equation (6) to explicitly account for the interaction between the evolving concentration and the pre-existing residual stress $\left(\sigma_{r,\varepsilon }\right)$ [1,17].

(15) $$\frac{\partial c_{k,c}}{\partial t}=\frac{\partial }{\partial x}\left[D_{k,c}\frac{\partial c_{k,c}}{\partial x}+\frac{D_{k,c}E_{c}V_{k,c}^{2}c_{k,c}}{9RT}\frac{\partial c_{k,c}}{\partial x}\left(\frac{\partial^{2}c_{k,c}}{\partial x^{2}}-\frac{9RT}{D_{k,c}E_{c}V_{k,c}^{2}c_{k,c}}\frac{\partial c_{k,c}}{\partial x}\left(\frac{\partial \sigma_{r,\varepsilon }}{\partial \varepsilon }\frac{\partial \varepsilon }{\partial t}+\frac{\partial \sigma_{r,\varepsilon }}{\partial c_{k,c}}\frac{\partial c_{k,c}}{\partial t}\right)\right)\right]$$

#### 4.3.1. Derivation of Diffusion-Induced Stress in the Bilayer

The diffusion-induced stress arises from the constraint imposed by the substrate on the coating’s swelling during diffusion. The derivation applies to the General Mechanical Framework (Equations (9)–(11)) defined in Section 4.3, using the chemical eigenstrain,

Chemical Eigenstrain and Stress

The unique input for this problem is the local volumetric expansion (chemical or swelling strain) produced by the insertion of diffusing species:

(16) $$\varepsilon_{\mathrm{ij}}^{\mathrm{chem}}\;=\;\frac{V_{k}c_{k}}{3}\;\delta_{\mathrm{ij}}$$

where ${\overset{ˉ}{V}}_{k}$ is the partial molar volume, $c_{k}$ is the species concentration, and $\varepsilon_{ij}^{chem}$ is the chemical eigenstrain. This strain serves as the input to the stress relation (Equation (8)), which is subsequently solved using the force and moment equilibrium conditions (Equations (9)–(11)).

Final Expression for the Diffusion-Induced Stress

Solving the system yields the strain field in terms of material constants, geometry, and mean concentrations $c_{k,c}$ and $c_{k,s}$. The resulting diffusion-induced normal stress in the coating is:

(17) $$\sigma_{d,c}\;=\;E_{c}\left[\frac{E_{c}hV_{k,c}c_{k,c}\;+{\;E}_{s}sV_{k,s}c_{k,s}}{3(E_{c}h\;+\;E_{s}s)}\;+\;(x-t_{b})\frac{2\left[E_{c}E_{s}\mathrm{hs}(h\;+\;s)(V_{k,c}c_{k,c}-V_{k,s}c_{k,s})\right]}{E_{c}^{2}h^{4}\;+\;E_{s}^{2}s^{4\;}+\;2E_{c}E_{s}\mathrm{hs}(2h^{2}\;+\;3\mathrm{hs}\;+\;2s^{2})}\right]-\frac{1}{3}c_{k,c}V_{k,c}$$

where $c_{k,c}$, $c_{k,s}$, ${\overset{ˉ}{V}}_{k,c}$, and ${\overset{ˉ}{V}}_{k,s}$ represent the concentrations and partial molar volumes of the diffusing species in the coating and substrate, respectively. Substituting Equations (9)–(11) into the general framework gives the solution for coating deformation, including the coupling effect of residual stress and diffusion-induced stress. The final term, $\frac{1}{3}\;{\overset{ˉ}{V}}_{k,c}c_{k,c},$ removes the free swelling contribution to obtain the constrained stress field [2,18,22,39,44,45,46,47].

#### 4.3.2. Feedback Diffusion Stress $\left(\sigma_{d\prime }\right)$

To determine the influence of the evolving concentration and residual stress on the generated stress, a feedback term $\left(\sigma_{d\prime }\right)$ is introduced. This term represents the rate of change in the diffusion stress over time, derived by solving the coupled diffusion Equation (8) [2,5,9].

The effect of residual stress and concentration of diffusing corrosive species on feedback diffusion-induced stress can be found by solving Equation (8) as:

(18) $$\sigma_{d}\prime \;=\;\frac{\partial \sigma_{d,c}}{\partial t}\;=\;\frac{\left(9\mathrm{RT}\;+\;E_{c}V_{k,c}^{2}c_{k,c}\right)\frac{\partial^{2}c_{k,c}}{\partial x^{2}}\;+{\;E}_{c}V_{k,c}^{2}{\left(\frac{\partial c_{k,c}}{\partial x}\right)}^{2}-\frac{9\mathrm{RT}}{D_{k,c}}\frac{\partial c_{k,c}}{\partial t}-D_{k,c}\frac{\partial \sigma_{r,c}}{\partial x}}{V_{k,c}^{2}\frac{\partial c_{k,c}}{\partial x}}$$

The feedback diffusion-induced stress $\left(\sigma_{d}^{\prime }\right)\;$ can be a uniform state of equibiaxial compression or tension, depending upon the concentration of diffusing corrosive species and the direction of the residual stress gradient. When under compression, the coating at cathodic sites de-bonds from the substrate, which, upon reaching the critical size, results in the formation of a blister as shown in Figure 3 [1,6,7].

#### 4.3.3. Derivation of Feedback Diffusion-Induced Pressure $\left(p_{d}\right)$

To link this internal stress to the blister propagation model (which requires an applied interfacial pressure), a conversion based on the hoop stress principle for circular geometries is applied. This principle relates to the internal pressure $\left(p\right)$ in a thin-walled structure to the stress $\left(\sigma \right)$, wall thickness $\left(h\right)$, and blister radius $\left(r\right)$ through [6,7]:

$$p\;=\;\frac{h\cdot \sigma }{r}$$

By substituting the analytical expression for the feedback diffusion-induced stress $\left(\sigma_{d}^{\prime }\right)$ from Equation (17) into the pressure conversion relationship, the final expression for the feedback diffusion-induced pressure $\left(p_{d}\right)$ acting within the delaminated blister cavity is obtained: $p_{d}=\frac{h}{r}\cdot \sigma_{d}\prime$

This leads directly to Equation (18) [4,5,18]:

(19) $$p_{d}\;=\;\frac{h}{r}\left[\frac{\left(9\mathrm{RT}\;+\;E_{c}V_{k,c}^{\;2}c_{k,c}\right)\frac{\partial^{2}c_{k,c}}{\partial x^{2}}\;+\;E_{c}V_{k,c}^{2}{\left(\frac{\partial c_{k,c}}{\partial x}\right)}^{2}-\frac{9\mathrm{RT}}{D_{k,c}}\frac{\partial c_{k,c}}{\partial t}-D_{k,c}\frac{\partial \sigma_{r,c}}{\partial x}}{{V\;}_{k,c}^{2}\frac{\partial c_{k,c}}{\partial x}}\right]$$

This pressure $\left(p_{d}\right)$ serves as the critical mechanical loading parameter for the von Kármán plate theory employed in the subsequent fracture mechanics analysis.

### 4.4. Normalisation and Final Propagation Criterion

The final propagation criterion is obtained by normalising the derived feedback diffusion-induced pressure $\left(p_{d}\right)$ with the critical buckling pressure $\left(p_{cr}\right)$, which represents the coating’s mechanical resistance to crack growth [10,11].

This normalisation modifies the conventional form of normalised stress for buckling $\left(\frac{\sigma }{\sigma_{\mathrm{cr}}}\right)$:

$$\frac{\sigma }{\sigma_{\mathrm{cr}}}\propto \frac{(1-\nu_{c}^{2})}{E_{c}}\sigma {\left(\frac{r}{h}\right)}^{2}$$

By substituting the complex diffusion and stress terms (derived for $\sigma_{d}^{\prime }\;$ and converted to $\left(p_{d}\right)\;$ and algebraically rearranging the constants, the ratio is transformed into a normalised pressure ratio, $p_{d}/p_{cr}$. This substitution modifies the geometric term from

$${\left(\frac{r}{h}\right)}^{2}\mathrm{to}{\left(\frac{r}{h}\right)}^{3}$$

This results in the final, coupled stability criterion (Equation (19)), which defines the condition for unstable blister growth when $\frac{p_{d}}{p_{\mathrm{cr}}}\;>\;1$ [2,9]:

(20) $$\frac{p_{d}}{p_{\mathrm{cr}}}=\frac{(1-\nu_{c}^{2})}{E_{c}}\left[\frac{(9\mathrm{RT}\;+{\;E}_{c}V_{k_{c}}^{\;2}c_{k_{c}}^{2})\frac{\partial^{2}c_{k_{c}}}{\partial x^{2}}\;+\;E_{c}{V\;}_{k_{c}}^{2}{\left(\frac{\partial c_{k_{c}}}{\partial x}\right)}^{2}-\frac{\partial c_{k_{c}}}{\partial t}\frac{9\mathrm{RT}}{D_{k_{c}}}-\frac{\partial \sigma_{r_{c}}}{\partial x}}{V_{k_{c}}^{\;2}\frac{\partial c_{k_{c}}}{\partial x}}\right]{\left(\frac{r}{h}\right)}^{3}$$

The ratio $p_{d}/p_{cr}\;$ can increase due to either an increase in the blister radius (r) or an increase in the feedback diffusion-induced pressure $\left(p_{d}\right).\;$ Fracture propagation is then governed by the resulting mode-adjusted driving force F, which depends on the elastic energy release rate (G) and a dimensionless mixed-mode function (f(ψ)). Although considerable progress has been achieved in the numerical modelling of cathodic blister evolution, the degree of experimental validation varies among the reported predictions. Experimental studies have generally confirmed the significant influence of modulus mismatch on interfacial stress distribution, coating delamination, and blister growth, and have also indicated that coating thickness ratio has a comparatively smaller effect on blister evolution than material properties and interfacial adhesion. However, predictions related to the critical blister radius and the transition from axisymmetric to non-axisymmetric blister morphologies are still supported primarily by analytical and numerical studies, with only limited experimental validation available. Furthermore, comprehensive experimental investigations under coupled diffusion, mechanical loading, and tribological conditions remain scarce. Future work should therefore focus on systematic experimental validation of these predictions under realistic service conditions to improve the reliability and applicability of advanced electro-chemo-mechanical and meso-mechanical models.

#### Derivation of Feedback Diffusion-Induced Pressure and Final Propagation Criterion

To link the internal stress to the blister propagation model (which requires an applied interfacial pressure), a conversion based on the hoop stress principle for circular geometries is applied. By substituting the complex analytical expression for the feedback diffusion-induced stress $\left(\sigma_{\mathrm{diff}}\right)$ into this conversion formula, the final expression for the feedback diffusion-induced pressure $\left(p_{d}\right)\;$ acting within the delaminated blister cavity is obtained. This pressure serves as the crucial mechanical load input for the von Kármán plate theory used in the subsequent fracture mechanics analysis.

The Interfacial Propagation Criterion (Equation (19)):

The final propagation criterion is obtained by normalising the derived feedback diffusion-induced pressure $\left(p_{d}\right)\;$ with the critical buckling pressure $p_{cr}$, which represents the coating’s mechanical resistance to crack growth. The fully coupled stability criterion establishes the condition for unstable blister growth when the normalised pressure ratio, $\frac{p_{d}}{p_{\mathrm{cr}}}$, exceeds unity when $p_{d}/p_{cr}>1$.

This criterion is defined by the analytical expression derived from the integration of the stress-coupled diffusion equation and the von Kármán plate theory:

(21) $$\frac{p_{d}}{p_{\mathrm{cr}}}=\frac{(1-\nu_{c}^{2})}{E_{c}}\left[\left(\frac{9\mathrm{RT}\;+\;E_{c}V_{k,c}^{2}c_{k,c}}{9\mathrm{RT}}D_{k,c}\frac{\partial^{2}c_{k,c}}{\partial x^{2}}\right)\;+\;E_{c}V_{k,c}^{2}{\left(\frac{\partial c_{k,c}}{\partial x}\right)}^{2}-\frac{\partial c_{k,c}}{\partial t}\frac{9\mathrm{RT}}{D_{k,c}}-\frac{\partial \sigma_{r,c}}{\partial x}V_{k,c}^{2}\frac{\partial c_{k,c}}{\partial x}\right]\frac{r}{h^{3}}$$

where the variables include

$p_{d}/p_{\mathrm{cr}}:$ The normalised pressure ratio, which acts as the blister driving force.

$D_{k,c}$: The diffusion coefficient of species k in the coating.

$V_{k,c}$: The partial molar volume of species k.

$\sigma_{r,c}:\;$The residual stress in the coating.

r and h: The blister radius and coating thickness.

This equation demonstrates that the driving force for propagation is directly proportional to the normalised diffusion-induced stress terms (inside the brackets).

### 4.5. Conceptual Origin of the Mode-Adjusted Driving Force F

Fracture propagation is ultimately governed by the mode-adjusted driving force (F), which is formulated to account for the mixed-mode (Mode I/Mode II) nature of interface delamination. The derivation generalises the Linear Elastic Fracture Mechanics (LEFM) criterion for mixed-mode failure:

In addition to LEFM, surface-based cohesive zone models have been widely adopted for analysing interface delamination in adhesively bonded polymer-coated steel structures, offering simplified parameter identification, efficient finite element implementation, and reduced computational cost while maintaining comparable predictive capability for interfacial failure [48,49]. LEFM Failure Criterion: In LEFM, crack propagation occurs when the total energy release rate (G) equals the critical interfacial fracture toughness $\left(\Gamma_{c}\right)$ [19,23]:

$$G\;\geq \;\Gamma_{c}$$

Mode-Dependent Toughness $\left(\Gamma_{c}\right)$: Experiments show that the critical fracture toughness $\left(\Gamma_{c}\right)$ is not a constant value but depends on the mixed-mode angle (ψ), with toughness typically increasing as the loading shifts toward Mode II (shear). This dependence is described by a function, $\Gamma_{c}(\psi ).$

Normalisation and Generalisation: To simplify the failure criterion, the Mode I toughness $(\mathrm{IC}\;\mathrm{or}{\;\Gamma }_{I})$ is chosen as a reference material constant. The mode-dependent toughness $(\Gamma_{c}\left(\psi )\right)$ is then expressed using the dimensionless mixed-mode function f(ψ):

$$\Gamma_{c}\left(\psi \right)\;=\;\frac{\mathrm{IC}}{f(\psi )}$$

Final Driving Force (F): By defining the mode-adjusted driving force (F) as the normalised energy release rate, the failure condition simplifies to a single-parameter comparison. The driving force F normalises the Elastic Energy Release Rate (G) by the dimensionless mixed-mode function (f(ψ)) [4,19,23]:

(22) $$F\;=\;\frac{G}{f(\psi )}$$

The blister propagation criterion is then stated as: F ≥ IC [4,19,23]. This approach allows the complex mode-dependent failure boundary to be incorporated into a single, unified driving force term  F.

The terms G and f(ψ) are defined by:

Elastic Energy Release Rate (G): This term is derived from the Von Kármán plate theory and quantifies the energy available for crack extension, considering bending and membrane forces.

(23) $$G=\frac{6(1-\nu_{c}^{2})}{E_{c}h^{3}}\left(M_{c}^{2}\;+\;\frac{1}{12}h^{2}VN^{2}\right)$$

Mixed-Mode Function f(ψ): This semi-empirical function is a fracture criterion that accounts for the increase in material toughness as the loading shifts away from pure Mode I toward shear-dominated (Mode II) conditions.

(24) $$f(\psi )={sec}^{2}[(1-\lambda )\psi ]$$

Here, ψ  is the mixed-mode ratio parameter defining the relative contribution of Mode II to Mode I fracture at the crack edge, whereas λ  is a material fitting parameter with a value of approximately 0.2 for the primer-coated steel system.

Mixed-Mode Ratio (ψ)

The ratio of the Mode II stress intensity factor ($K_{2}$) to the Mode I stress intensity factor ($K_{1}$) determines the mixed-mode behaviour.

(25) $$\psi =\frac{K_{2}}{K_{1}}=cot\left(\frac{cos\;\omega +\left(\frac{h\nabla N}{\sqrt{12M_{c}}}\right)sin\;\omega }{\left(\frac{h\nabla N}{\sqrt{12M_{c}}}\right)cos\;\omega -sin\;\omega }\right)$$

The parameter ω  in this equation depends on Dundurs’ elastic mismatch parameter $\left(\chi \right)$, where $\chi =\frac{{\overset{ˉ}{E}}_{c}-{\overset{ˉ}{E}}_{s}}{{\overset{ˉ}{E}}_{c}+{\overset{ˉ}{E}}_{s}}.$

Blister Height and Mechanical Coupling

(26) $$\frac{h\nabla N}{\sqrt{12M_{c}}}=0.2(1+\nu_{c})\left(\frac{\delta }{h}\right)$$

Coupling to Diffusion-Induced Pressure

(27) $$\frac{\delta }{h}=\sqrt{\frac{1}{0.2(1+\nu_{c})+0.2(1-\nu_{c})\left(\frac{p_{d}}{p_{cr}}-1\right)}}$$

The normalised blister height δ/h provides the crucial connection to the normalised diffusion-induced pressure $\;p_{d}/p_{cr}$. The final condition for incipient blister propagation requires the mode-adjusted driving force F to equal the critical Mode I interfacial toughness $\left(I_{C}\right)$:

(28) $$F=I_{C}$$

By integrating the analytical expressions from the diffusion model (Equation (17)) into the mechanical driving force (Equation (18)), this framework provides a unified predictive criterion for coating degradation

(29) $$\sqrt{\frac{G_{0}}{F}}=p_{d}\sqrt{\frac{(1-\nu_{c})h}{E_{c}I_{C}}}$$

where $G_{0}$ is equivalently expressed as:

(30) $$G_{0}=p_{d}^{\;2}\frac{(1-\nu_{c})h}{E_{c}}$$

Equation (30) provides the incipient-spread criterion, establishing the threshold condition for blister propagation in terms of the normalised diffusion-induced stress, $\frac{\sigma_{d}^{\prime }}{\sigma_{cr}},$ or equivalently, $\frac{p_{d}}{p_{cr}}.$

This criterion provides the theoretical basis for predicting coating failure associated with blister propagation.

## 5. Numerical Method

A structured, nonlinear, time-dependent computational framework, consistent with established approaches in stress-assisted diffusion and thin-film buckling mechanics, is used to solve the coupled diffusion–mechanics system that governs bilayer blister evolution [18,20,27,28,29,30,31,32]. In the second-order finite difference scheme that discretises the coating thickness, the spatial domain is divided into N nodes along the x-direction. The temporal evolution is conducted through an unconditionally stable implicit time-marching method, which is a standard technique for solving nonlinear diffusion problems with chemical expansion and stress feedback involved [23,32,41]. This has the benefit of accurately resolving the coupled concentration–stress evolution even in the steep gradients that are characteristic of blister growth.

Boundary conditions are subjected to the same physical restrictions as the polymer–metal bilayers are. At the coating surface (x = h), a constant concentration of species is imposed, representing the continuous ingress of the electrolyte near the blister cavity, which is like the conditions used in cathodic delamination studies [2,5,7,12]. At the coating–substrate interface (x = 0), the zero-flux boundary condition ensures that the metallic substrate is impermeable, and this is consistent with the classical corrosion-driven blister transport models [9,20,23]. Such conditions permit the simulation of corrosive environments that are representative of and consistent with the experimentally reported scenarios of blistering, as shown in Figure 3 [1].

The two numerical strategies employed are sequential coupling and fully coupled iteration.

In the sequential method, first, the diffusion equation is solved using the stress field from the previous time step. The resulting concentration profile is then supplied to the mechanical solver for the update of diffusion-induced stresses through the chemical eigenstrain formulation [20,27,28,41]. This method reduces computational costs while retaining strong coupling fidelity.

In the fully coupled solution, concentration and stress fields are solved together within each iteration. Although this method is more computationally intensive, it is suitable for dealing with strong nonlinear interactions, especially when the mechanical response has a considerable effect on the local concentration gradient [24,32,42].

The convergence of the nonlinear system is assured by iterating until the differences in the updates of both the concentration and stress fields are smaller than a specified tolerance, usually <10^6^, which is in accordance with the practices used in nonlinear thin-film and diffusion-stress simulations [30,42,43].

The numerical framework can depict wear profiles and kinetics that are observed in experiments [8]. Kinetics of normalised wear depth are shown to decrease with increasing number of fretting cycles; whereas, wear profiles that are normalised have U-shaped distributions and maximum wear in the middle of the interface. There are different coatings that are characterised by different degradation rates; for example, Ni/ZrO_2_ has the highest wear depth, followed by Ni/SiC, Ni/Al_2_O_3_, and Ni/GPL. The contact shape transitions from elliptical to Hertzian and finally to quasi-flat, which reflects the mechanisms of micro-cutting, micro-delamination, and micro-ploughing. By considering these characteristics of wear, the model mimics the physically recognisable degradation pattern under cyclic loading, which increases its predictive capacity for tribological applications.

A systematic mesh-refinement study has been done, which guarantees that the numerical solution is not affected by the spatial discretisation artefacts. It is confirmed that mesh convergence has been achieved when refinement does not affect the stress concentration gradients or blister pressure evolution. The numerical framework gives us transient concentration fields, the evolution of residual, diffusion-induced, and total stress distributions, and the corresponding feedback diffusion-induced pressure applied to the coating–substrate interface. The overall result of these findings is a solid and repeatable computational device for evaluating the blister’s initiation, growth, and stability kinetics, thus enlarging the predictive power of meso-mechanics-based blister models as presented in the Table 2 below [20,21,25,43].

### Quantitative Validation: The Critical Stability

The integrated meso-mechanics model has been confirmed by the predicted stability condition being compared with the experimental observations of transitions in blistering for polymer–metal bilayers [5,18,20,21,23]. A linear stability analysis provides a simplified formula for the critical blister radius that separates the stable axisymmetric deformation from the unstable propagation. The approximate expression for the critical radius is:

$$r_{c}\;\approx \;\kappa D\sigma_{\mathrm{eff}}^{1/2}$$

D is the flexural rigidity of the coating.

$\sigma_{\mathrm{eff}}$ is the effective compressive stress (residual + diffusion components) acting on the coating.

κ is a dimensionless factor dependent on the mode number and elastic mismatch [21,23,26,46].

Prediction–Experiment Agreement:

$r_{c}$ matches the values observed in experiments for the beginning of blister instability, thereby confirming the correctness of the mechanical–chemical coupling mechanism [18,21,25,51].

Axisymmetric to Non-Axisymmetric Transition:

When r > $r_{c}$, compressive stresses destabilise the axisymmetric blister, triggering the classical telephone-cord buckling transformation described in thin-film buckling literature [14,23,26,29].

Parametric Sensitivity:

Sensitivity studies reveal that $r_{c}$ depends strongly on the elastic modulus ratio Ec/Es, confirming mechanical mismatch as the dominant stability-governing parameter, consistent with analytical buckling predictions [16,21,25]. The predictions concerning blister instability and stress evolution in coated layers can be associated with the same or similar phenomena happening in the case of gear systems and other tribological contacts, where the rolling and sliding loads produce residual stresses that, in turn, are responsible for the wear and propagation of fatigue cracks.

## 6. Results and Discussion

The assessment of the proposed meso-mechanics model’s predictive performance and mechanistic correctness is done through a comparison of its output with analytical predictions and experimental data reported in the literature on blistering, fracture, and stress-assisted diffusion [16,17,18,19,20,21,22,23,24,25,26,27,28,29,30]. The tribological performance of gears, where rolling and sliding contacts lead to the generation of residual stresses, fatigue cracks, and wear, is the context in which these findings are to be interpreted further.

### 6.1. Validation Against Nazir et al.

As the initial step, the model validation is conducted in conjunction with the original meso-mechanics-based approach of the Khan–Nazir approach (2016) [20]. For the same material, geometric, and environmental conditions, the predicted blister radius, peak interfacial pressure, and species concentration profiles in time show remarkably close agreement with the reference model. Stress distribution profiles display limited differences due to the inclusion of higher-order curvature terms, neutral-axis shifts, and a fully formulated diffusion-induced stress based on Larché–Cahn theory [8,9,24]. These refinements contribute to physical fidelity and localisation of the stress fields more accurately than the thin-film delamination experiments [18,23,47] reflect.

### 6.2. Residual Stress Fields

The computed residual stress distribution reflects the combined effects of the mismatch in thermal expansion, the difference in modulus between coating and substrate, and the bending due to curvature, consistent with the bilayer mechanics formulations [16,22,27]. The higher-order bending terms lead to even better agreement with analytical predictions for curved bilayers, and they also capture the tensile–compressive zones known to promote interfacial decohesion [19,21,25]. This improved representation is essential for accurately predicting blister nucleation sites and the early stages of damage evolution.

### 6.3. Diffusion-Induced Stress Fields

One of the sources of diffusion-induced stresses is the chemical eigenstrain caused by the penetration of corrosive species, which is a process well documented in the literature on stress-assisted diffusion [8,9,24,32]. The results indicate that the stress not only depends on but is also strongly influenced by local factors such as concentration gradients, partial molar volume, and the substrate that imposes the local constraint. Through the model, it is revealed that the build-up of compressive stress at the cathodic sites facilitates the process of debonding and cavity formation, thereby reinforcing the mechanisms which have been reported in cathodic blistering experiments [1,5,12,20].

### 6.4. Pressure Evolution

One can say that the diffusion-induced stresses converted to effective interfacial pressure give an understanding of the mechanical driving force for blister propagation in a direct manner. The simulation indicates an initial quick pressure rise along with the concentration field evolution, which is then followed by a pressure plateau that is determined by coating rigidity and substrate compliance [18,26,30]. Unlike many previous models that take blister pressure as an input [17,19], the present one derives itself consistently from the interaction of mechanical and chemical processes, which is in line with experimentally observed pressure–time responses of polymer–metal blisters [6,10,43].

### 6.5. Critical Blister Radius

The stability analysis indicates a critical radius for the blisters, which, when exceeded, will lead to the loss of stability of the axisymmetric blister and its transformation into a non-axisymmetric cord-like morphology. The predicted values are in close agreement with experimental measurements and classical analysis of buckling for thin films on soft substrates [14,21,22,23,26,29]. Furthermore, the critical radius is found to be extremely sensitive to elastic mismatch, which is in line with the theory of fracture and buckling mechanics [16,22]. Additionally, the flexibility of the coating seems to play a role in determining when instability will occur.

### 6.6. Parametric Studies

The results of a thorough parametric study illustrate the power of the key parameters to shape:

Thickness Ratio:

Coating-to-substrate thickness ratio variations have a minor impact on blister propagation, and these results are in line with previous analytical and experimental studies [17,19,23].

Modulus Ratio:

Elastic mismatch is a powerful factor influencing stress partitioning, the build-up of interfacial pressure, and the size of critical blisters, which in turn confirms predictions from classical delamination mechanics [16,22].

Partial Molar Volume:

Higher values cause an increase in chemical eigenstrain, and thus the diffusion-induced stress is at a higher level, which is in line with the models of stress-assisted diffusion [8,9,24].

Species Concentration:

A greater concentration creates more pressure and faster blistering, which agrees with experimental results on the kinetics of cathodic delamination [1,5,12].

Curvature:

Residual curvature changes the areas of tensile/compressive stress and affects the paths of nucleation and growth, thus corroborating results from studies on the buckling of thin films [21,26].

### 6.7. Improved Physical Interpretation

The collective meso-mechanics approach unveils the mechanism more efficiently than the prior semi-empirical blister models [17,19,21]:

The great dependence on modulus ratio is due to the shifting of stress between coating and substrate, and it is thus explained that coatings with mechanical softness are more likely to suffer blistering.

The little influence of the thickness ratio is attributed to the limitation of stress evolution that is imposed by geometric constraints during debonding.

The interaction between chemical eigenstrain, residual stress, flexural rigidity, and feedback pressure explains the previously unaccounted experimental trends in blister growth and morphology [18,20,21,23].

## 7. Outlook for Gear Tribology and Future Work

The study’s conclusions support the application of meso-mechanics-based blister modelling to gear tribology, where rolling and sliding contacts, residual stresses and fatigue phenomena all have a major impact on the lifespan of the components. Although the existing model is particularly good at blister formation, development and stability in coating–substrate systems, there are still a lot of unexplored avenues in moving gear systems from coating–substrate modelling to practical applications.

Key Research Gaps and Future Directions:

Contact Mechanics and Residual Stresses:

The integration of rolling contact fatigue (RCF) mechanics into the meso-mechanics framework is the next goal. The surfaces of gear teeth are constantly loaded and stressed in different directions, which causes small cracks to form and then to grow. Using contact mechanics models that consider Hertzian stress distributions, surface roughness, and lubrication effects will make it possible to make a valid assessment of the changes in residual stress and the propagation of fatigue cracks in both coated and uncoated gear materials.

Materials Selection:

The tribological loading in each case is determined largely by the influences of the gear materials and coatings. The ability of the meso-mechanics model to predict outcomes will be instrumental in material choice, i.e., substrates like metals, coatings like polymers, and hybrid systems. The future also involves compatibility assessment in terms of both mechanical and chemical factors, taking diffusion-induced stresses and environmental degradation into consideration. Additionally, regulatory criteria such as REACH and RoHS standards will be factored in to ensure that the materials suggested are not just eco-friendly but also safe for industry.

Residual Stress and Surface Engineering:

The factory, surface treatment and operational loading-induced residual stresses have a decisive role in coating delamination and crack fatigue initiation. The future work will yield the quantification of the combination of thermal, mechanical, and diffusion-induced stresses with surface topography and microstructural heterogeneities. This approach will help optimise surface engineering strategies such as shot peening, nitriding, and thin-film deposition to enhance fatigue resistance in gears.

Friction, Wear, and Corrosion Interactions:

The wear, friction, and corrosion processes proceed in harmony and together form the wearing of materials, which happens in machines, particularly in gear systems under rolling–sliding conditions. The meso-mechanics model will be developed further to combine the simulations of mechanical and chemical degradation. Experimental wear kinetics, frictional heating, and chemical attack will be integrated for a predictive service-life estimation of coatings and substrates under operational conditions.

Numerical and Simulation Enhancements:

Future research will be based on the current finite-difference framework and move to high-fidelity numerical simulations through finite element and multi-scale modelling to capture the complexities of non-axisymmetric blister morphologies and real-world gear contact scenarios. The simulations will incorporate residual stress fields, diffusion-induced stresses, mixed-mode fracture criteria, and cyclic loading effects that will create a comprehensive tool for coating design and failure prediction.

To sum up, the meso-mechanics framework extension to gear tribology will allow mechanistically informed estimates of residual stress, fatigue crack initiation, wear, friction, and corrosion. Hence, it will be easier to make the optimal choice of materials and coatings under the actual industrial conditions while ensuring their compliance with the regulations. This integration not only helps in predicting the behaviour of tribo-mechanical components but also strengthens the knowledge of blister mechanics together with the practical design and durability issues in the case of gears and other tribologically loaded components.

## 8. Conclusions

This review examined the current state of knowledge on cathodic blister evolution in multilayer coating systems by synthesising developments in diffusion-assisted degradation, stress evolution, interfacial fracture mechanics, and coating delamination. The reviewed literature demonstrates that the mechanism of cathodic blister formation is governed by the coupled interaction of diffusion, residual stress development, and fracture behaviour rather than by any individual process. Meso-mechanics-based models, together with electro-chemo-mechanical and phase-field approaches, have significantly advanced the understanding of blister initiation, propagation, and coating failure under corrosive environments.

Recent developments in multilayer coating architectures, substrate deformation analysis, polymer microstructural design, and numerical modelling have further improved the ability to assess coating durability under realistic service conditions. The reviewed studies indicate that multilayer coatings can effectively reduce stress concentration and delay crack propagation, while advanced polymer systems with enhanced molecular architectures improve crack resistance and interfacial stability. These advances provide valuable insight into the design of more durable protective coating systems for aggressive environments.

This review also highlights that cathodic blistering should be considered within the broader framework of tribological and corrosive degradation. In many engineering applications, including gears, bearings, marine structures, and automotive components, rolling/sliding contact, residual stress evolution, wear, and tribo-corrosion interact with diffusion-driven processes to influence blister initiation, coating delamination, and long-term structural integrity. Considering these mechanisms together provides a more comprehensive understanding of coating degradation under practical operating conditions.

Despite the significant progress achieved in recent years, several challenges remain. Existing models require further experimental validation, particularly under combined mechanical and electrochemical loading conditions. Future research should focus on integrating multiscale numerical modelling with experimental investigations, advanced multilayer coating architectures, smart polymer materials, and environmentally sustainable material selection. Such developments will improve the prediction of coating degradation, enhance coating durability, and support the development of more reliable protective coating systems for tribological and corrosive applications.

## Figures and Tables

**Table 1:** Post-2016 advances in meso-mechanics-based cathodic blister modelling and related polymer coating studies.

Year	Model/Study	Key Advancement	Relevance to Blister Prediction
2016	Nazir et al. [1]	Classical meso-mechanics model combining diffusion and interfacial fracture.	Provides the baseline framework for predicting blister nucleation and growth.
2016	Xu et al. [6]	Investigates the stabilisation mechanisms of secondary nuclei during lamellar polymer-crystal growth.	Provides fundamental insight into polymer crystallisation and lamellar growth; its relevance to coating blister prediction is indirect.
2018	Latif et al. [33]	Prognostic life-assessment model for multilayer coating systems using solid mechanics, fracture, and diffusion.	Relates blister propagation and interfacial debonding to coating service-life prediction.
2018	Nazarov et al. [34]	Used scanning Kelvin probe measurements to assess corrosion and interfacial deadhesion beneath an epoxy barrier coating on steel.	Demonstrates how undercoating corrosion promotes loss of adhesion, providing insight into the interfacial processes preceding blister formation
2021	Effendy et al. [35]	Nonlinear modelling of blister growth dynamics.	Extends blister evolution analysis beyond classical linear formulations.
2023	Hoseinpoor et al. [36]	Investigates the effects of mechanical deformation on coating damage, water uptake, and blistering.	Demonstrates that deformation increases coating permeability and water uptake, thereby increasing susceptibility to corrosion-induced blistering.
2024	Guo et al. [37]	Experimentally investigates the coupled effects of high hydrostatic pressure and cathodic polarisation on epoxy-coating failure.	Demonstrates that hydrostatic pressure accelerates water uptake and structural degradation, while cathodic polarisation promotes interfacial delamination.
2025	Liu et al. [38]	Combined laser thermal-shock experiments, finite-element modelling, and a cohesive-zone model to analyse multimodal crack growth in functionally graded thermal-barrier coatings.	Shows how coating-gradient design and crack morphology distribute interfacial stresses, reduce the energy-release rate, and improve resistance to delamination.

**Table 2:** Comparison of existing blister models (from Nazir et al., 2016 [1]).

Model/Author	Main Mechanism	Key Parameters	Strengths	Limitations
Xu et al. (2003) [42]	Axisymmetric blister delamination	Load, blister radius, film thickness	Closed-form analytical solution	No diffusion–stress coupling
Prawoto and Onn (2012) [50]	Diffusion-driven blister formation	Diffusivity, concentration, time	Models ionic transport	No coupled fracture mechanics
Suo (2003) [43]	Failure mechanics of layered films	Residual stress, elastic mismatch, toughness	General reliability framework	No electrochemical effects
Hutchinson & Suo (1992) [23]	Mixed-mode interfacial cracking	Energy-release rate, mode mixity, toughness	Foundation of blister fracture models	No corrosion or diffusion effects
Thouless (1990–2000) [14]	Thin-film adhesion fracture	Toughness, residual stress, crack driving force	Strong fracture-mechanics basis	No chemically driven blistering
Nazir et al. (2016) (Reference model) [1]	Coupled diffusion, residual stress and fracture	Diffusivity, concentration, residual stress, fracture energy	Coupled chemo-mechanical model	Requires complex numerical implementation.

## Data Availability

No new data were created or analysed in this study. Data sharing does not apply to this article.
